# Complex Clinical Interplay: A Case Report of Systemic Lupus Erythematosus Coexisting With Type II Cryoglobulinemia

**DOI:** 10.7759/cureus.76969

**Published:** 2025-01-05

**Authors:** Munsef Barakat, Madison Ladines, Salem Vilayet, Vishwajeeth Pasham, Tibor Fulop

**Affiliations:** 1 Nephrology, Medical University of South Carolina, Charleston, USA; 2 Medicine, College of Medicine, Medical University of South Carolina, Charleston, USA; 3 Pathology and Laboratory Medicine, Medical University of South Carolina, Charleston, USA

**Keywords:** class iv lupus nephritis, glomerulonephritis, systemic lupus erythema, therapeutic plasmapheresis, type 2 cryoglobulinemia

## Abstract

Systemic lupus erythematosus (SLE) is a multi-faceted autoimmune disease with diverse clinical manifestations, often diagnosed through specific immunological markers. Another noteworthy immunological phenomenon associated with SLE is cryoglobulinemia (CG), characterized by circulating immunoglobulins that precipitate at lower temperatures. Although the overlap of SLE and CG is documented, its precise prevalence remains elusive. This paper presents a unique case of a patient diagnosed with lupus cryoglobulinemic glomerulonephritis through biopsy. The patient attained full remission after an initial induction using the Eurolupus protocol complemented with plasmapheresis, with subsequent maintenance therapy managed via rituximab.

## Introduction

Systemic lupus erythematosus (SLE) is a prevalent autoimmune disease characterized by multi-system involvement. Predominantly affecting young females, its incidence is notably higher among African Americans and individuals of Asian descent [1]. The clinical manifestations of SLE are multifaceted, varying based on the organ system impacted. This ranges from skin rashes and photosensitivity to non-destructive arthritis. However, the disease can also give rise to more severe complications, including pericarditis, myocarditis, renal implications, and central nervous system involvement. The diagnosis of SLE is supported by the presence of specific immunological markers, such as antinuclear antibodies (ANA), double-stranded (dsDNA) antibodies, and low complement levels.

Cryoglobulinemia (CG), another notable immunological manifestation, refers to the presence of circulating immunoglobulins that precipitate at reduced temperatures. It is traditionally classified into three types. Type I, or simple CG, consists solely of a monoclonal immunoglobulin and is primarily associated with hematological malignancies like lymphoma, lymphocytic leukemia, and myeloma. Conversely, types II and III, collectively termed mixed CG, incorporate polyclonal IgG and monoclonal IgM immunoglobulins or entirely polyclonal immunoglobulins, respectively. Despite the frequent association of CG with hepatitis C infections, it is also observed in SLE patients. Indeed, SLE is prominently ranked among autoimmune diseases co-existing with CG, sharing this distinction with Sjogren’s syndrome and certain hematological malignancies [2].

The clinical presentation of CG is predominantly small vessel vasculitis, affecting skin, kidneys, and peripheral nerves, often culminating in sensorimotor polyneuropathy. Vascular damage in type I arises mainly due to the precipitation of the abnormal globulin. In contrast, vasculitis observed in types II and III stems from the deposition of immune complexes [3,4].

The intersection of CG and SLE is well-documented across various literature, including case series. However, its exact prevalence in SLE patients remains somewhat elusive. Some studies, like one by García-Carrasco M et al., have estimated this figure to be as high as 25% [5]. Generally, SLE is more prone to induce mixed CG than type I. A retrospective evaluation involving over 200 SLE patients discerned the presence of CG in a substantial 66% of them, with type III being the most common [6].

In this context, our case spotlights a patient diagnosed with lupus cryoglobulinemic glomerulonephritis via biopsy. Following an initial induction with the Eurolupus protocol combined with plasmapheresis, the patient attained full remission. Maintenance therapy was subsequently achieved using monoclonal anti-CD 20 therapy with rituximab.

Parts of this project have been presented at the ASN Kidney Week 2023 Annual Meeting, Nov 01-04, 2023, Philadelphia, PA, USA. 

## Case presentation

A 32-year-old African American female presented with an acute onset of shortness of breath, chest tightness, and progressive lower extremity swelling. Concurrently, she developed blisters on both ankles that evolved into ulcers. She denied joint pain, fever, chills, oral or nasal ulcers, or any changes in the color or consistency of urine.

Approximately one month before her presentation, she experienced a persistent cough and chest discomfort. Despite seeking medical care and receiving a brief antibiotic course, there was no notable amelioration. Her medical history included SLE, myositis, and type II CG. Importantly, she had no prior history of lupus nephritis and had never required a renal biopsy before her current presentation. Her medications on admission included azathioprine (150 mg daily), hydroxychloroquine (200 mg daily), prednisone (5 mg daily), and methotrexate (10 mg weekly) - the latter having been reduced from 20 mg weekly dose due to asymptomatic transaminitis the previous month.

In addition, she had a known history of B-cell lymphoproliferative disease with monoclonal IgM gammopathy spikes for several years but with no discernible end-organ involvement. Twice she underwent bone marrow biopsies, which revealed only mild plasmacytosis. Given the lack of symptoms or significant biopsy anomalies, she was maintained under surveillance only.

Initially, she had sought care at another institution. However, due to deteriorating respiratory function and the onset of acute kidney injury (AKI) coupled with progressive oliguria, she was transferred to our facility. Upon arrival, her clinical assessment revealed a state of acute illness marked by tachycardia, respiratory distress characterized by tachypnea and shallow breaths, hypoxic respiratory failure, and mild lower limb pitting edema. The patient also exhibited jugular venous distension. Vital signs were recorded as follows: heart rate: 124 bpm, blood pressure: 150/90 mmHg, and oxygen saturation maintainable: >90% via nasal cannula. Owing to her respiratory distress, she alternated between non-invasive ventilation (NIV) and high-flow nasal cannula and had one instance of hemoptysis.

Laboratory investigations (Table 1) on admission highlighted leukocytosis, normocytic anemia, and elevated total creatine kinase (CK) levels, consistent with her myositis history. Renal indices were perturbed, indicating AKI with raised serum creatinine, proteinuria, hematuria, and oliguria. The autoimmune profile was positive for ANA but negative for anti-dsDNA antibodies. Complement levels were suppressed. Serological tests for hepatitis B surface antigen, hepatitis C antibodies, and HIV antibodies were all negative. However, serum CG was qualitatively positive, and the rheumatoid factor level was elevated (365.3 IU/mL; reference range <30 IU/mL). Arterial blood gas on 100% O_2_ showed the following: pH: 7.31, PO_2_: 109, PCO_2_: 23, HCO_3_: 22, with an oxygen saturation (O_2_sat) of 98%. 

**Table 1:** Initial relevant laboratory values on admission.

Blood work on admission	Value	Reference range
Serum sodium	140 mmol/L	135-145mmol/L
Serum potassium	5.2 mmol/L	3.5-5.1 mmol/L
Serum chloride	108 mmol/L	98-108 mmol/L
Serum bicarbonate	20 mmol/L	22-29 mmol/L
Blood urea nitrogen	74 mg/dL	7-21 mg/dL
Serum creatinine	2.7 mg/dL	0.6-1.1 mg/dL
B-type natriuretic peptide (BNP)	2476 pg/mL	<100.0 pg/mL
White cell count	15,000 K/cumm	4.00-11.00 K/cumm
Hemoglobin	8.8 g/dL	11.0-15.0 gm/dL
Platelets	332 K/cumm	140-440K/cumm
Procalcitonin	0.1 ng/mL	≤0.10 ng/mL
Antinuclear antibodies	Positive 1:1280	Negative
double-stranded DNA antibodies	Negative	Negative
Phosphorus	6.9 mg/dL	2.3-4.7 mg/dL
Total serum calcium	6.7 mg/dL	8.4-10.3 mg/dL
Serum magnesium	2.1 mg/dL	1.6-2.6 MG/DL
Total creatine kinase	6177 U/L	20-190 U/L
Alanine transaminase (ALT)	171 U/L	5-45 U/L
Aspartate transferase (AST)	149 U/L	5-34 U/L
Serum albumin	2.6 g/dL	3.5-5.0 g/dL
Alkaline phosphatase	52 U/L	35-150 U/L
International normalized ratio (INR)	1.1	0.90-1.20
Thyroid-stimulating hormone (TSH)	0.2 mIU/L	0.35-4.94 mIU/L
Free T4	0.76 ng/dL	0.70-1.48 ng/dL
Complement C3	50.1 mg/dL	82-193 mg/dL
Complement C4	2.9 mg/dL	15-57mg/dL

Urine analysis was significant for hematuria, indicated by a presence of 180 RBCs/HPF, with negative nitrite, ketones, and an insignificant count of WBCs. The urine protein to urine creatinine ratio was elevated at 3338 mg/g.

A transthoracic echocardiography demonstrated a normal left ventricular ejection fraction of 54%, with no evidence of regional wall motion abnormalities. Notably, there was a moderate circumferential pericardial effusion, but sonographic signs of cardiac tamponade were absent.

Due to the patient's oliguria, evidence of AKI, hematuria, and pertinent medical history, a decision was made to perform a percutaneous renal biopsy on the fourth day of her admission.

**Figure 1:**
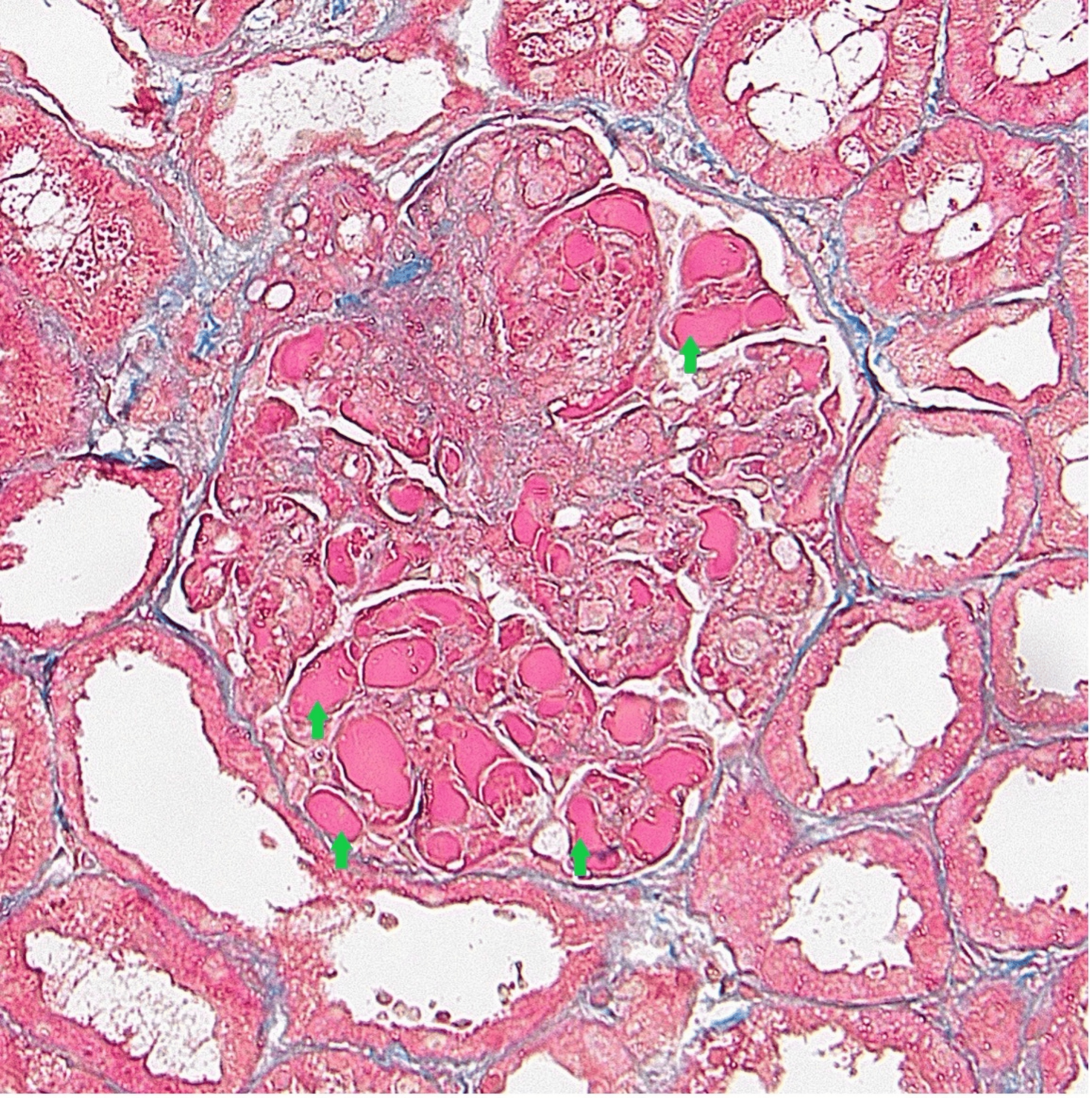
Trichrome stain demonstrating prominent hyaline pseudo-thrombi within capillary loops (green arrow).

**Figure 2:**
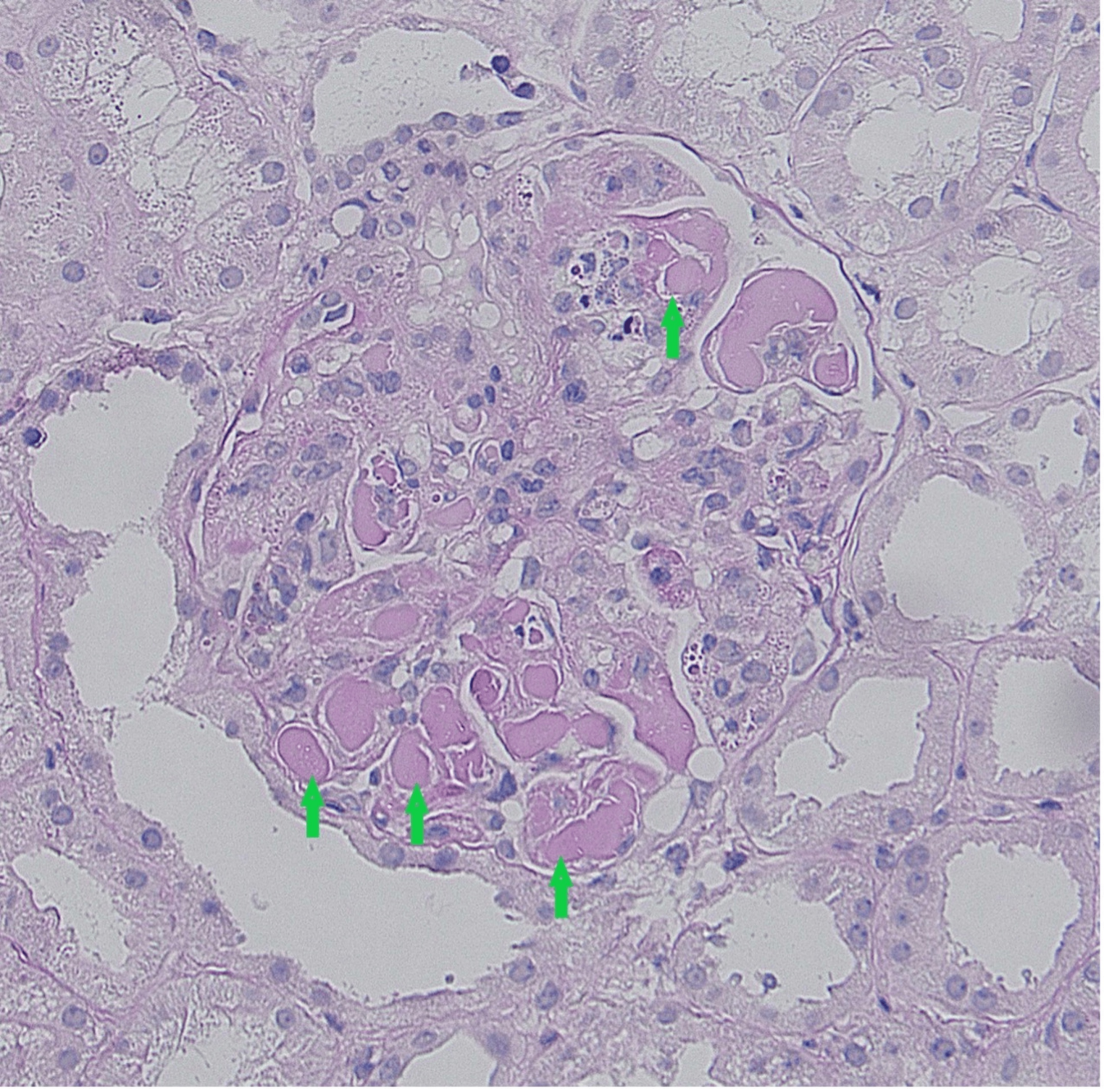
Strongly PAS-positive prominent hyaline pseudo-thrombi within the capillary loops (green arrow). PAS, periodic acid–Schiff

Histological examination of the kidney biopsy revealed 45 glomeruli per section, of which two had global sclerosis. All glomeruli were characterized by pronounced hyaline pseudo-thrombi (Figure 1 and Figure 2). Occasional double contouring of the glomerular basement membrane was observed on the silver stain, but there were no signs of endocapillary proliferation, necrosis, or crescents. The silver stain showed no spikes. The renal interstitium was spared from fibrosis or tubular atrophy. Additionally, the renal arteries were free from fibroelastic intimal thickening or arteriolar hyalinosis. The Activity Index was noted as 3/24, attributed to capillary wall changes, while the Chronicity Index stood at 1/12 due to glomerular sclerosis. Immunofluorescence staining revealed positivity in the capillary lumina for IgG (3+), IgA (3+), IgM (4+), C3 (3+), C1q (4+), kappa (4+), and lambda (4+). In contrast, it was negative for fibrin.

**Figure 3:**
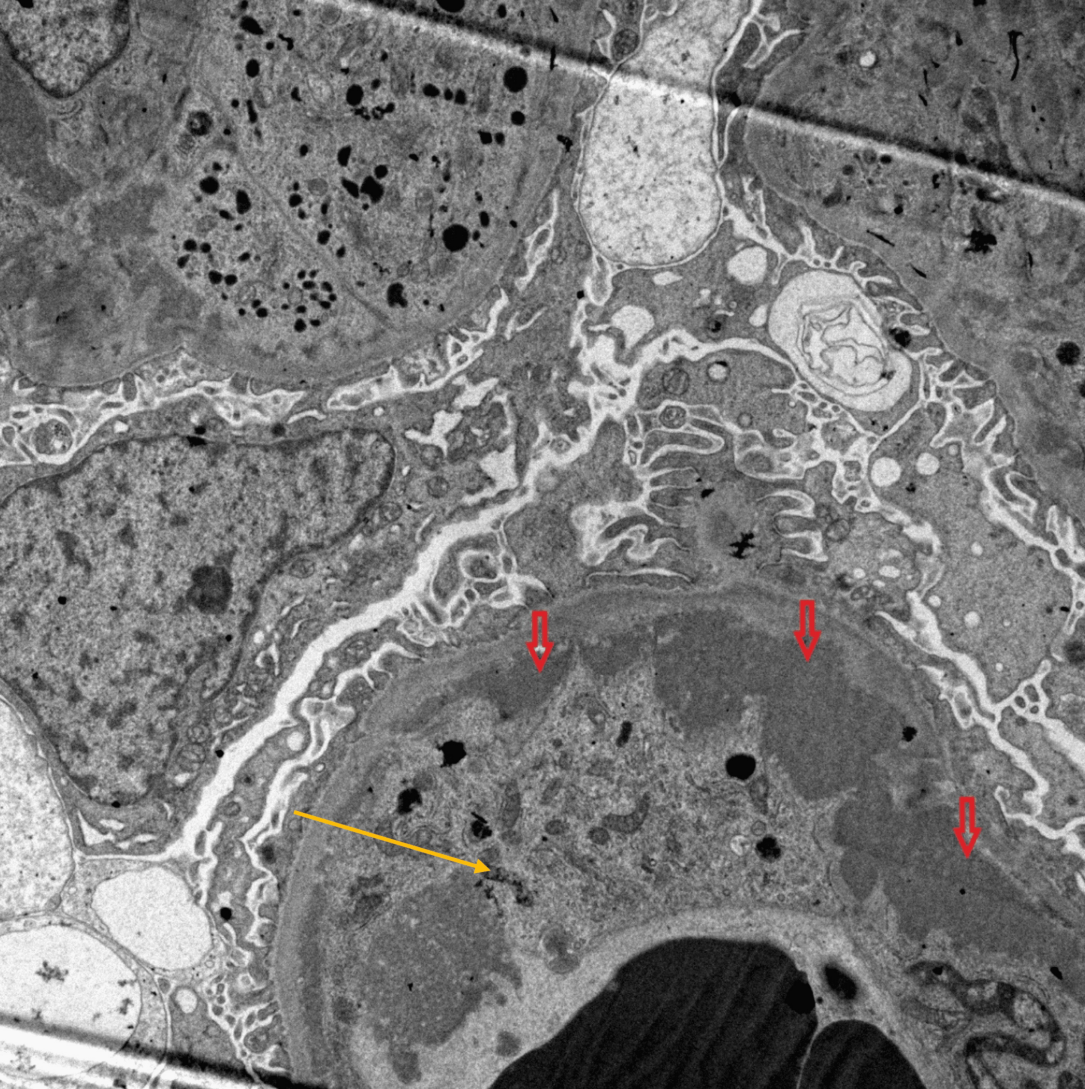
Electron microscopy shows prominent subendothelial deposits (red arrow) and a macrophage with phagocytosed cryoglobulins (note the microtubular ultrastructure) (yellow arrow).

Electron microscopy findings included partial effacement of the visceral epithelial cell foot processes (less than 25%). Electron-dense material, in some areas, showcasing a microtubular appearance, filled certain capillary loops. Subendothelial and mesangial deposits were present (Figure 3). Additionally, some capillary loops contained macrophages with electron-dense phagolysosomes. The glomerular basement membrane in certain loops exhibited reduplication with the interpositioning of mesangial cell cytoplasm. Although the endothelial cells appeared swollen, no tubuloreticular inclusions were observed.

Considering the patient's clinical presentation, her medical background, and the biopsy results, the findings were deemed most consistent with lupus cryoglobulinemic glomerulonephritis as opposed to isolated lupus nephritis.

The patient commenced treatment following the Eurolupus protocol, which involved cyclophosphamide 500 mg administered intravenously every two weeks for a period of three months. Alongside this, she received pulse steroids and subsequently transitioned to a steroid taper. Owing to the renal involvement manifesting as CG, therapeutic plasma exchange (TPE) with 5% albumin and fresh frozen plasma (FFP) was initiated, with the patient undergoing nine sessions over a span of three weeks.

Subsequent to the completion of cyclophosphamide therapy, the patient was shifted to a maintenance regimen involving the anti-CD 20 monoclonal antibody, rituximab 1 g for two doses, and later kept on regular rituximab every six months. Hydroxychloroquine dosage was adjusted to 200 mg daily, and a tapering dose of prednisone starting at 60 mg daily was administered.

During subsequent follow-up consultations, the patient exhibited normalized renal function and achieved complete remission. This was evidenced by the resolution of proteinuria, which has been sustained for five years.

## Discussion

The etiology of lupus nephritis is rooted in immune intolerance toward autoantigens, subsequently leading to immune system activation. It is well-understood that those afflicted with lupus nephritis bear both a genetic predisposition and an environmental trigger component [7,8]. 

The concurrent occurrence of SLE and CG is not uncommon, though CG often remains asymptomatic [9,10]. In our case, clear evidence of cryoglobulinemic arteritis/vasculitis and glomerulonephritis was discernible from the renal biopsy, which is believed to be a primary contributor to the AKI, rather than mere lupus nephritis.

In a retrospective study evaluating over 200 SLE patients without concurrent hepatitis C infection, CG was identified in 66% of subjects. Among these, type III CG was more prevalent than type II. Interestingly, this study found no disparity in the incidence rates of lupus nephritis between patients solely with SLE and those with both SLE and positive CG [6].

Treatment strategies for non-infectious CG are determined by the cause and the degree of organ involvement. In scenarios involving significant vasculitis events and major organ involvement, a combined approach utilizing multiple immunosuppressive agents, such as steroids, cyclophosphamide, and rituximab, becomes pivotal [11,12]. TPE has a broad range of indications and is used in a multitude of clinical scenarios [13,14]. In this specific case, only the removal of abnormal cryoglobulin was necessary and enabled an expeditious “debulking” of abnormal immunoglobulin and mitigated risk factors for ongoing renal insult. With 5% albumin and FFP replacement fluid every other day, we have not encountered a bleeding problem; to be noted, TPE with FFP is immunosuppressive on its own [13].

It is noteworthy that our patient never previously exhibited lupus nephritis, nor did she ever require a renal biopsy given her consistently normal renal parameters. Although she was diagnosed with type II CG due to an underlying lymphoproliferative disorder, she remained asymptomatic without discernible end-organ manifestations. Given the flare-up of SLE and unchecked autoimmunity, it is postulated that a concomitant manifestation of lupus nephritis and active cryoglobulinemic vasculitis arose, as evidenced by the renal biopsy. Finally, this case confirmed the invaluable contribution of kidney biopsy to expedite diagnosis and optimize therapy in clinical dilemmas [15]. As such, the incorporation of plasma exchange in the treatment protocol was deemed appropriate and likely to have brought incremental benefits to the case.

## Conclusions

SLE and CG often coexist, but the clinical significance and interplay between these conditions, particularly in the context of renal involvement, remains a complex subject. Although adding TPE to standard medical therapy has not been shown to have any benefits in cases of pure lupus nephritis, it still has its role in cases of refractory disease, coexisting catastrophic antiphospholipid syndrome, diffuse alveolar hemorrhage, or, in our case, coexisting with CG.

This case underscores the importance of distinguishing between the effects of pure lupus nephritis and those complications introduced by CG, especially when they jointly contribute to an acute renal injury. While both SLE and CG have unique pathways of renal damage, their concomitant presence may exacerbate renal injury, necessitating a more multifaceted therapeutic approach. Our therapeutic intervention, which combined the Eurolupus protocol with plasma exchange, showcases the potential benefits of an integrated treatment strategy for such complex clinical presentations. Further studies are warranted to optimize the management of patients with concurrent SLE and CG, ensuring the best possible outcomes.
